# Challenges for Antepartum Care of the Individual with Perinatal Substance Use: An Empirical Integrative Review of Novel Approaches to Improve Care

**DOI:** 10.1111/jmwh.13714

**Published:** 2024-11-27

**Authors:** Lauren Taylor Narbey, Alice Curtis Cline

**Affiliations:** ^1^ University of Pittsburgh, School of Nursing Pittsburgh Pennsylvania

**Keywords:** antepartum care, pregnancy complications, substance use disorders including tobacco

## Abstract

**Introduction:**

Perinatal substance use continues to rise across the United States presenting unique challenges to providing antepartum care. Polysubstance use, limited and late engagement in health care, co‐occurring mood disorders, and several social barriers are well documented. This review seeks to summarize these barriers and present novel approaches to caring for this high‐risk population.

**Methods:**

Inclusion criteria for this study focused on peer‐reviewed articles that explicitly detailed a direct impact on the provision or receipt of antenatal care in the setting of substance use within the United States that were published in the last 5 years. PubMed and Web of Science were used to find applicable articles. Of the 156 articles found, 10 relevant articles were selected for the final empirical integrative review that entailed data evaluation using the Mixed Methods Appraisal Tool (MMAT) and thematic analysis.

**Results:**

10 review articles met inclusion; 3 were qualitative, 6 were quantitative and nonrandomized, and one was quantitative descriptive. Six articles met MMAT quality criteria, and there were significant limitations in every article. Topics included opioid use disorder (n = 6), general substance use (n = 3), and tobacco use (n = 1). Themes included integrated models of prenatal care, colocated care, resource coordination, and peer support along with the role of the perinatal health care professional and consistent use of a substance use screening tool.

**Discussion:**

A comprehensive and multidisciplinary care model is necessary to meet the complex and urgent needs of individuals with perinatal substance use that not only meets recommendations for opioid maintenance therapy or substance use cessation but the important areas of accessibility and interpersonal support. Future research should focus on the development, implementation, and evaluation of new models of care for this vulnerable population.

## INTRODUCTION

Rates of substance use in pregnancy have been rising in the last decade. A recent survey of substance use among adults estimates 8% to 11% of pregnant people report using at least one substance, including nicotine products, alcohol, cannabis, and illegal substances.^1^ Importantly, opioid use during pregnancy has increased by 400%,^2^ which has led to rising rates of relapse and overdose in the months following birth. In fact, opioid overdose is among the most common causes of maternal mortality in the first year after birth, nearly doubling between 2018 and 2021.^3^, ^4^, ^5^ Additionally, rates of polysubstance abuse, defined as opioid use plus at least one additional substance, are similar in pregnancy to the general population.^6^

Compared with the general population, individuals with perinatal substance use are much less likely to present for antepartum care.^1^, ^7^, ^8^, ^9^ Those that do often present late to health care in the second or third trimester, causing delays in obtaining the standard perinatal laboratory screenings and fetal ultrasound assessments. Peacock‐Chambers et al found that when individuals did engage with care, they were particularly in need of providers who were able to achieve collaborative engagement that included both support for them as a parent and person in recovery, as well as provision of support and resources to meet their complex needs.^10^ However, perinatal care providers have reported a lack of knowledge, resources, and legal ability to adequately meet the needs of these individuals.^11^, ^12^, ^13^, ^14^, ^15^

Continuing education (CE) is available for this article. To obtain CE online, please visit http://www.jmwhce.org. A CE form that includes the test questions is available in the print edition of this issue.

Provision of adequate, evidence‐based, holistic care for pregnant persons with substance use is thus essential for improving health during pregnancy, birth, and beyond. However, the barriers to receiving adequate care are well documented and include lack of clinician experience, challenges to care coordination, and stigma.^14^, ^16^, ^17^

The Substance Abuse and Mental Health Services Administration (SAMHSA) has established guidelines on the antepartum care of individuals with perinatal opioid use disorder (OUD). These recommendations include screening all pregnant persons for substance use with a validated tool, prescribing opioid maintenance therapy (OMT) when indicated, providing behavioral health care services, and planning for intrapartum pain management.^1^ For pregnant individuals with perinatal OUD, having access to providers who can prescribe OMT is essential to optimize maternal and infant health and prevent perinatal mortality and morbidity.^18^

**QUICK POINTS**

✦ Care models that integrate antenatal care, medication‐assisted treatment for substance use disorder, and behavioral health are more successful.
✦ Social support from peers and professionals is beneficial to individuals with substance use disorder during pregnancy.
✦ Group care models can be used to combine integrated care and peer support.
✦ All pregnant individuals should be consistently screened for substance use with a validated instrument throughout pregnancy.

The purpose of this review, therefore, was to explore the available evidence on the design of antenatal care for individuals with substance use disorder. We aim to summarize best practices in caring for pregnant clients and families experiencing substance use disorder to better prepare clinicians who are caring for this vulnerable population.

## METHODS

This empirical integrative review was conducted in line with best practice recommendations for conducting narrative reviews (of which an integrative review is a subtype) outlined by Ferrari^19^ and narrative synthesis guidelines developed by Popay et al.^20^ An empirical integrative review design was selected because of the diverse study designs of the publications available on the topic of antepartum care for individuals with perinatal substance use. Empirical integrative reviews allow for the analysis and synthesis of studies with diverse methodologies and can deliver a general overview of the best available evidence in an area of practice.^21^

### Search Strategy

Following the methodologic framework developed by Whittemore and Knafl^22^ and updated recommendations by Ferrari,^19^ 2 databases were selected for searches: PubMed and Web of Science. These databases include the majority of medical, nursing, and social science publications in the United States relevant to this topic. Search terms were developed after careful review of the literature and several pilot searches with the support of a library scientist. Medical Subject Heading terms were used when searching PubMed, and search terms were converted to keywords for Web of Science. Search terms were *quality of healthcare*, *maternal health services OR perinatal care*, and *substance‐related disorders OR substance use*. We included original articles with various methodologies: observational studies, experimental studies, and expert opinion. Studies were included if they were published in English within the last 5 years in the United States to reflect practice innovations based on the most up to date literature on perinatal substance use. Studies were also included if they examined prenatal care models or features and focused on a population of pregnant individuals with substance use disorders.

**Figure 1:**
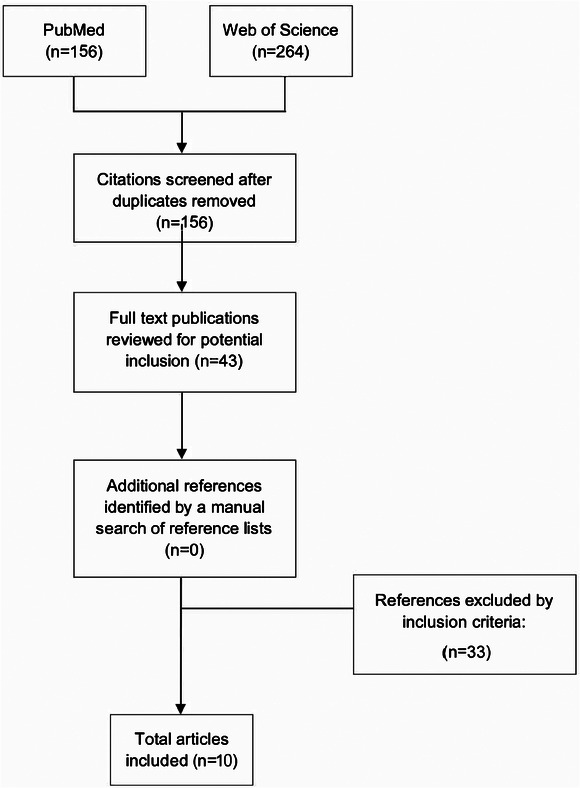
Preferred Reporting Items for Systematic Reviews and Meta‐Analyses Flow Chart of Literature Selection Process

The initial searches were conducted by LN. A total of 264 articles were identified. After duplicates were removed (n = 108), titles and abstracts were reviewed, and 43 articles then reviewed in full text. A total of 10 articles met inclusion criteria for this narrative review. Authors LN and AC independently assessed all abstracts and full text manuscripts. There were no disagreements on article inclusion. Preferred Reporting Items for Systematic reviews and Meta‐Analyses (PRISMA) guidelines were used for reporting the findings.^23^ See Figure 1 for the article selection flowchart using PRISMA guidelines.^24^

### Quality Evaluation

Author L.N. assessed the 10 included articles using the Mixed Methods Appraisal Tool (MMAT),^25^ which uses a table to evaluate quality in mixed methods studies. A version of the table Part I: MMAT, version 2011, was used for this study (Appendix S1).^26^ Using these guidelines, L.N. evaluated each article and identified any missing criteria. Author A.C. reviewed and agreed with the MMAT assessment for each included article.

### Analysis

Thematic analysis was conducted using both inductive and deductive coding. Coding was done independently by both authors by creating a table of the selected articles. All novel approaches used in the study were coded with memos on significant outcomes. The authors met and discussed their coding and memos in which codes were grouped into themes. Any disagreements were discussed and resolved by making amendments to code groupings and themes, respectively (Table 1 and Table 2).

**Table 1:** Description of Included Articles, Number of Participants, Model Used, and Main Findings

Author, Year	Approach or Model	Setting	N	Structure	Effect of Model
Boden et al, 2021^34^	Serial screening	Community hospital or clinic	251	The Boden self‐reported perinatal substance use screening tool was implemented during the first, second, and third trimesters.	Those who were completed the screening earlier and during each subsequent trimester were less likely to continue substance use or have a newborn be diagnosed or need treatment for NOWS.
Crane et al, 2019^30^	MOMS	4 site types in Ohio: community behavioral health, residential behavioral health, hospital‐based obstetric practice, and colocated obstetric and behavioral health	252 intervention, 846 comparison	MOMS participants received access to OMT through a MMH model that coordinated behavioral health and prenatal care with social supports for pregnant women with OUD enrolled in Medicaid in 1 of 4 site settings.	Increased OMT in all 3 trimesters. Increased uptake of behavioral health services in the second and third trimester. Continued OMT through 6 months postpartum. Lower out‐of‐home infant placement. Sites with colocated obstetric and behavioral health saw greater uptake of OMT and behavioral health services.
Fallin‐Bennett et al, 2020^27^	Peer support specialists for OUD	Outpatient clinic	9	Clients received care at a clinic with peer support.	Participants reported that their peer support specialist helped them feel supported during pregnancy and the postpartum period, were most helpful when the peer was authentic, supportive, and held the person accountable. Participants particularly appreciated feeling validated and receiving anticipatory guidance throughout the perinatal period.
Fallin‐Bennett et al, 2019^31^	Perinatal wellness navigator	Clinic at an academic medical center	50, 42 with OUD	1. One‐on‐one tobacco treatment 2. Comprehensive assessment of cessation barriers 3. Linkage to clinical or social services	Participants experienced reductions in number of cigarettes smoked per day, nicotine dependence, as well reductions in perceived stress and depression.
Goodman et al, 2019^32^	Perinatal checklist	8 sites in New England		1. A 26‐item checklist 2. A year‐long learning collaborative including at least one representative from each practice 3. Toolkit for the perinatal care of women with substance use disorders	Statistically significant increase in the number of records that correctly diagnosed substance use, access naloxone, prescribed nicotine replacement, counseling on breastfeeding benefits, and follow‐up Hepatitis C testing.
Goodman et al, 2022^35^	Integrated obstetric and substance use treatment	Rural	225	Patients received either coordinated, colocated obstetric care and OUD treatment (integrated treatment), or routine care (nonintegrated).	Those in integrated care were less likely to give birth prematurely, their infants had shorter hospitalizations, and lower rates of positive maternal urine toxicology screens at the time of birth.
Ostrach and Leiner, 2019^28^	Comprehensive outpatient perinatal substance use treatment program	Rural	27	Patients in this program receive OB and substance use treatment in integrated outpatient setting with the provision of wrap‐around services.	Participants reported valuing the supportive, nonjudgmental care they experienced in the program; discussed ambivalence or hesitation in starting or remaining on OMT because of stigma, fear of legal involvement social services, fear of withdrawal once they could no longer afford their medication once their health insurance was discontinued postpartum.
Paterno et al, 2019^33^	EMPOWER	Rural	38	Patients who screen positive for OUD are referred to an EMPOWER nurse coordinator who provides an education consultation and any needed referrals, including a recovery coach. Integrated OB care Third trimester nurse consultation that included creation and submission of a birth plan that can be submitted to social services	Increased breastfeeding initiation, continuation, and exclusivity Increased birth weight Increased referrals and access to support services, peer support being the most common
Short et al, 2024^39^	Group prenatal care for OUD	Academic medical center, urban	15 treatment, 56 comparison	CenteringPregnancy (group prenatal care) combines medical assessment of traditional antepartum care with comprehensive prenatal health education, consultation, and peer support facilitated by a credentialed health care practitioner.	Satisfaction with group prenatal care was high, with 100% reporting that they would recommend the program. Although statistical tests were not performed, a higher percentage of those in group care initiated breastfeeding and were still breastfeeding at discharge, received the Tdap vaccine, attended postpartum visits, and they reported fewer postpartum depression symptoms.
Sutter et al, 2019^29^	Group prenatal care for OUD	Urban university‐affiliated perinatal substance abuse treatment program (cares for approximately 200 women per year)	Not specified	Group prenatal care with behavioral health and optional prenatal yoga; team‐based, interprofessional.	Women are more likely to self‐disclose social needs and accept support after establishing more trusting relationships with group facilitators. Other themes or effects included continuity of care, trust, social connectedness, and high satisfaction and engagement.

Abbreviations: EMPOWER, Engaging Mothers for Positive Outcomes with Early Referrals; MMH, maternal medical home; MOMS, Maternal Opioid Medical Support; NOWS, neonatal opioid withdrawal syndrome; OB, obstetric; OMT, opioid maintenance therapy; OUD, opioid use disorder; Tdap, tetanus, diphtheria, and pertussis.

**Table 2:** Mixed Methods Appraisal Tool Evaluation of Included Articles With Interventions Targeting Prenatal Care and Substance Use

Author, Year	Met MMAT^25^ Criteria	Substance	Care Feature and Summary
Boden et al, 2021^34^	No, no mention of confounders	All	Early and serial screening for perinatal substance use
Crane et al, 2019^30^	Yes	Opioids	Integrated care: access to OMT through an MMH model that coordinated behavioral health and prenatal care with social supports
Fallin‐Bennett, 2019^31^	Yes	Tobacco	Care management: one‐on‐one tobacco treatment with comprehensive assessment of cessation barriers and linkage to clinical or social services
Fallin‐Bennett, 2020^27^	Yes	Opioids	Peer support: clients received care at a clinic with peer support
Goodman et al, 2019^32^	No	Opioids	Checklist: 26‐item checklist developed from a year‐long learning collaborative
Goodman et al, 2022^35^	Yes	Opioids	Integrated care: clients received either coordinated, colocated obstetric care and OMT (integrated treatment) or routine care (nonintegrated)
Ostrach and Leiner, 2019^28^	Yes	Opioids	Integrated care: clients receive OB care and OMT in an integrated setting with wrap‐around services
Paterno et al, 2019^33^	No	Opioids	Care management: nurse coordinator provides education, referrals, and entry into integrated OB care
Short et al, 2024^39^	Yes	Opioids	Group prenatal care
Sutter et al, 2019^29^	No	Opioids	Group prenatal care

Abbreviations: MMAT, Mixed Methods Appraisal Tool; MMH, maternal medical home; OB, obstetric; OMT, opioid maintenance therapy.

## RESULTS

An overview of the 10 manuscripts is presented in Table 1. Of the included articles, 3 were qualitative.^27^, ^28^, ^29^ Four were the reports of quality improvement projects or intervention pilots.^30^, ^31^, ^32^, ^33^ The remaining 3 were retrospective cohort descriptive studies.^34^, ^35^, ^36^ Six articles met all MMAT quality appraisal criteria (see Table 1).^37^ Several articles did not specifically account for confounders in their final analysis, and one lacked clarity on data collection methods. Eight studies focused specifically on OUD,^27^, ^28^, ^29^, ^30^, ^32^, ^33^, ^35^, ^38^ one addressed tobacco use,^31^ and one addressed general substance use.^34^ Five studies focused on the design of prenatal care and substance use recovery (integrated care^28^, ^30^, ^35^ and group care models^29^, ^38^). Two studies focused on care coordination or navigation.^31^, ^33^ One study each examined the effects of serial and early screening for substance use^34^ and the role of peer support^27^ respectively. After coding and discussion of themes, 3 primary themes emerged: (1) integrated models of antepartum care, (2) the role of the perinatal health care provider, and (3) the role of substance use screening throughout the perinatal period.

### Integrated Models of Prenatal Care

Several studies focused on the design and coordination of care delivered in the perinatal period.^28^, ^29^, ^30^, ^31^, ^33^, ^35^, ^39^ A major barrier commonly cited to receiving optimal care in the antepartum period was the lack of integration for the services and resources individuals require. In the traditional model of care, individuals must separately schedule and use services from perinatal care providers, addiction medicine specialists or OMT appointments, behavioral health care practitioners, and coordinate social or wrap‐around services such as transportation. There were several approaches detailed by these studies featuring colocated health care, resource coordination, and peer support.

#### Colocated Care

Three studies reported on the effects of integrating obstetric care, treatment for OUD, behavioral health care, and social services in a single facility or program.^28^, ^30^, ^35^ Although each article provided limited details on the clinical aspects of the development and implementation of these programs, all 3 found improvements in acceptability, accessibility, usage, and coordination of care in integrated models. Two additional studies occurred in the setting of integrated care but specifically focused on the inclusion of a group prenatal care model, with one study using the CenteringPregnancy program.^39^ Within the context of an integrated care program, group care contributed to further improved client outcomes.^29^, ^39^ Goodman et al found that pregnant clients in their integrated program had lower rates of premature births, shorter newborn hospitalizations, and lower rates of positive urine toxicology screens at the time of birth.^35^

When behavioral health care services were integrated in peripartum care, individuals had higher rates of perinatal care usage, including receiving adequate prenatal care, attending behavioral health care visits, receiving OMT, and attending pediatric visits.^30^ In the study on smoking cessation, participants who received behavioral health support reported greater success with smoking cessation, lower rates of stress and postpartum depression, and higher rates of referrals to other needed services.^31^

#### Resource Coordination

Provision of care coordination, specifically wrap‐around or social services, proved essential to supporting the complex needs of this client population. Crane et al^30^ specifically discussed the role of their program in improving interactions with child welfare services and referrals for individuals using a managed care plan. They included child welfare personnel in their program development and trainings, which increased client engagement in prenatal care.^40^ Community or social support workers were able to help clients successfully enroll in supplemental nutrition programs, coordinate transportation, obtain health insurance, and secure safe and affordable housing. They were able to support clients in navigating appropriate follow up for additional health care services such as referrals and appointments with maternal fetal medicine for high‐risk health issues, treatment for Hepatitis C, and consultations with lactation specialists or neonatology.^28^, ^30^, ^35^

#### Peer Support

Both formal and informal peer support appears to have an impact on prenatal outcomes. Participants reported especially valuing support from “someone's that's been there, lived it, seen it.”^27^ Peers helped individuals avoid relapse and were most effective when they could be authentic, supportive, and hold them personally accountable.^27^ Studies of group prenatal care models, in which participants serve as peer supporters to their group members (informal support), reported increased rates of breastfeeding, vaccine administration, and postpartum visit attendance.^39^ Paterno et al reported that formal peer support was the most common resource requested by clients.^33^

### The Role of the Perinatal Health Care Professional

Physicians, midwives, and nurses providing perinatal care can have a substantial positive impact on the prenatal care received by this population. Given appropriate counseling by health care professionals, participants reported feeling validated specifically when receiving anticipatory guidance and valued supportive, nonjudgmental care.^28^, ^37^ This was often achieved when providers extended appointment times and used active listening to better understand the challenges that individuals were experiencing. This led to increased trust and engagement, improved prenatal care adherence, and increased resilience for relapse.^29^ Participants were also more likely to accept and use referrals when they had a trusting relationship with their health care providers.^29^

Most studies described a process by which health care professionals were educated and trained to care for individuals with perinatal substance use. Topics included group prenatal care with CenteringPregnancy,^41^ initiation and maintenance of OMT, approaches to harm reduction, provision of naloxone, tobacco and cannabis cessation, Hepatitis C screening and diagnosis, integration of peer support into prenatal care, substance use screening, neonatal opioid withdrawal syndrome education, and the treatment of mood disorders. These trainings were delivered through case conferences, learning collaboratives, and provision of evidence‐based templates for use in electronic medical systems. Several studies went beyond education and training and provided health care professionals with tools to implement the education that they received through the provision of guidelines and algorithms, integration of recommendations into medical records, and technological assistance.^28^

### The Role of Substance Use Screening

Although only one article focused explicitly on the impact of substance use screening, all included articles described substance use screening as a vital and necessary part of their program's structure or means of assessing continued or new substance use throughout the pregnancy. Two articles discussed screening methods and tools in detail, one using the 4 P's Plus (Parents, Peers, Partner, Past, Pregnancy)^42^ and the other creating tools combining the tolerance, worry about drinking, eye‐opener, amnesia, and cut down on drinking (TWEAK: Tolerance, Worry, Eye Opener, Amnesia, Cut Down)^43^ and cutting down, annoyance by criticism, guilty feeling, and eye‐openers (CAGE: Cut, Annoyed, Guilty, and Eye)^44^ approaches. The 4P's screens individuals with 5 specific questions related to substance use in themselves as well as those in their social circle. TWEAK focuses on at‐risk drinking in the perinatal period, whereas CAGE screens for alcoholism. Boden et al created a screening tool (Boden Screening Tool) based on the TWEAK and CAGE tools and a self‐report that encompassed all legal and illegal substances.^45^ Of particular importance, they found that earlier and repeated screenings were associated with decreased substance use.

## DISCUSSSION

The barriers facing provision of antepartum care for individuals with perinatal substance use are many. Social stigma, lower socio‐economic status, lower education, limited access to transportation, polysubstance use, and co‐occurring mood disorders have been well documented.^14^, ^46^, ^47^, ^48^ However, improving care for these families is imperative to improving outcomes not only for the pregnant individual, but for their families as well.^49^

Providing high quality, comprehensive, integrated, and colocated antepartum and substance use recovery care has been shown in the majority of studies in this review to be both effective and acceptable. Integrated care programs require significant infrastructure to design, implement, and evaluate, especially if a group prenatal care model is used. Goodman et al detail the development of a 26‐item checklist for best practice in caring for individuals and families dealing with perinatal substance use.^32^ This multisite, multistate, well‐funded effort was supported by multiple stakeholders over several years. This same approach was used in Ohio with similar funding, length of time commitment, and needed infrastructure.^30^ Funding sources could include payer models such as fee‐for‐services from insurance companies and Medicaid, government funding, research grants, philanthropic initiatives, or a combination of sources.^14^ Development of such robust programs, particularly in rural communities, may still prove difficult. Telehealth has shown promise for recovery services and lactation support and may help support and supplement antepartum care and the provision of OMT.^50^, ^51^

Although having integrated and colocated prenatal care, substance use treatment, behavioral health care, and social services is optimal, authors of the studies included in this review offered few details on how these services were implemented clinically. Articles described integrated models that were developed to work within the constraints of the geographical, funding, and policy differences encountered at each location.^14^ This makes it challenging to offer guidance on best practices and share information with colleagues who are looking to create their own programs.

Given the high incidence of mood disorders among individuals with perinatal substance use, meeting the mental and social needs of this population is paramount.^9^, ^52^, ^53^, ^54^, ^55^ Peer support and behavioral health care services especially in the form of group prenatal care may be helpful in supporting pregnant individuals with substance use. Fallin‐Bennett et al discussed the importance of peer support from those who have lived experience with perinatal substance use.^27^ Support from peer doulas with training in substance use recovery support has been shown to be beneficial in the intrapartum and early postpartum period. Training for doulas in this setting may include building skills as health care advocates, improving maternal health literacy, and guidance on how to promote self‐advocacy for clients. Using this support in antepartum care may amplify the impact.^56^ Training for health care professionals is paramount to supporting individuals with substance use, which can include education on prescribing OMT, the usage and application of substance use, as well how to appropriately manage pain for perinatal individuals who are opioid tolerant.^27^

The American College of Obstetricians and Gynecologists and the SAMHSA recommend universal substance use screening with a validated tool for all pregnant individuals at least once during each trimester.^1^ Little is known about when screenings are being administered, how they are being documented in the medical record, or what referrals or services are being provided in the event of a positive screen. This is important given the stigma, fear, and legal implications of documented substance use during pregnancy. Many states continue punitive legal consequences for pregnant individuals who screen or test positive, which has shown to increase risks for both the pregnant person and their newborn.^57^ Consistent screening with clear planning and communication with the health care team are necessary to help individuals navigate their recovery during pregnancy in a nonjudgmental and supportive manner. Universal drug testing is not recommended, and confirmative substance use testing, usually through a urine drug screen, is only recommended when indicated to assess for continued recovery or active illicit substance use.^15^, ^58^, ^59^, ^60^ On a policy level, states with punitive mandated reporting measures have worse outcomes for families on OMT. These measures should be removed, and policies should shift to focus on providing evidence‐based treatment.^40^

Policy makers and stakeholders such as insurance companies must remove all barriers to continued access to OMT. All perinatal care providers are on the frontlines of primary perinatal health care and in ideal position to provide integrated and colocated services. Although federal regulations have relaxed for buprenorphine prescribing, state operating procedures have not necessarily followed suit, particularly in allowing advanced practice providers such as midwives to prescribe OMT.^40^, ^61^ Full recognition and reimbursement for all services provided in an integrated model is both complex and necessary to facilitate optimal care for this vulnerable population who face significant barriers to health care service engagement.^9^, ^13^, ^14^, ^40^, ^62^, ^63^, ^64^

### Implications for Practice

Continued education, collaboration, and training for health care professionals is an important piece of providing client‐centered, holistic care for this vulnerable and often stigmatized population.^62^, ^65^, ^66^ Ongoing access to resources and support for health care providers may improve prenatal outcomes for individuals with perinatal substance use. However, providing comprehensive and up to date information and tools is often met with many challenges, including limited funding and time available for professionals to complete the education.^67^, ^68^, ^69^ Although not explicitly required, perinatal care providers should receive training specifically for the prescription of OMT for their clients,^70^, ^71^, ^72^ be provided protected time in which to complete the education, and face no barriers to prescribing OMT.^40^ Obtaining a waiver to prescribe buprenorphine is no longer required;^73^ however, the DEA does require a one‐time 8‐hour training for safe opioid prescribing and substance use education before practitioners can apply for or renew their DEA license.^74^

Creating a client‐centered, collaborative team approach with input from invested perinatal health care professionals and community stakeholders has shown to improve outcomes for clients and may facilitate cost and resource sharing.^11^, ^54^, ^75^ Establishing these teams takes substantial effort, funding, and administrative support. Grant and institutional funding may be needed to support the establishment of collaborative structures, including the integration of care planning into the electronic health record. Health care professionals can also play a pivotal role in decreasing stigma experienced by their clients during their prenatal care experience. Using client‐centered language such as *opioid use disorder* or *substance free* can remove some of the shame and guilt pregnant individuals experience when presenting to care.^53^, ^76^ Clear, evidence‐based policies and practices that are discussed with each individual regarding substance use screening and mandated referrals are incredibly important. This includes appropriate informed consent for testing with consistent counseling on the risks, benefits, and limitations of each test.^53^, ^58^, ^77^

### Strengths and Limitations

A strength of this review is that it includes studies with a variety of methodologies, including both qualitative and quantitative methods. Designs include descriptive studies, retrospective cohort, and pilot evaluation. Pilot evaluations are especially valuable to this topic because they report on the benefits and barriers to implementation in vivo.

Limitations include that several of the studies failed to meet quality metrics when assessed with the MMAT instrument. Many of the studies were quite small, including fewer than 50 participants, which limits generalizability. Although the inclusion of such varied methodologies provides breadth in our review, it also contributes to challenges with comparison. Additionally, our search strategy used only 2 search engines to a single string of search terms and to publications in the last 5 years. We included only studies conducted in the United States and published in English. This limits external validity because the US health care system is unique in its state‐specific public and private design. Some findings may be generalizable to other health care systems, although they may only be relevant in specific jurisdictions.

Intervention research in the perinatal population faces many ethical limitations, so it is rare to find a randomized control trial. In many cases, we are limited to descriptive or quasiexperimental studies. Empirical integrative reviews are inherently prone to bias, but they allow authors to synthesize research and generate recommendations for future research and changes in clinical practice.^19^

## CONCLUSION

High quality, comprehensive perinatal care is essential for keeping individuals with perinatal substance use and their families well and safe through the perinatal period. The personal and system barriers this population experiences are well documented. Solutions to these problems are less well‐studied. Health care systems need to be reimagined to incorporate perinatal care, substance use treatment, mental health treatment, social services, care coordination, and peer support. Novel health care approaches may optimize perinatal care to pregnant women with substance use by improving the identification of individuals in need, reducing barriers to health care, increasing the scope of health care, and improving the quality of health care. Health care professionals and health care systems must increase substance use education and training on providing high quality care to this vulnerable population, by following national and professional society guidelines.

## CONFLICT OF INTEREST

The authors have no conflicts of interest to disclose.

## Supporting information




**Appendix S1**. Mixed Methods Appraisal Tool, version 2018.^25^
